# Association of Body Mass Index with Insulin-like Growth Factor-1 Levels among 3227 Chinese Children Aged 2–18 Years

**DOI:** 10.3390/nu15081849

**Published:** 2023-04-12

**Authors:** Yang Li, Xinnan Zong, Yaqin Zhang, Jiayun Guo, Hui Li

**Affiliations:** 1 Department of Growth and Development, Capital Institute of Pediatrics, Beijing 100020, China; 2 Peking Union Medical College, Chinese Academy of Medical Sciences, Beijing 100730, China; 3 Department of Endocrinology, Genetics and Metabolism, Beijing Children’s Hospital, Capital Medical University, National Center of Children’s Health, Beijing 100045, China

**Keywords:** insulin-like growth factor-1, body mass index, underweight, obesity, children

## Abstract

Objectives: Insulin-like growth factor-1 (IGF-1) levels are affected by nutritional status, yet there is limited research exploring the association between body mass index (BMI) and IGF-1 levels among children. Methods: This cross-sectional study included 3227 children aged 2–18 years without specific diseases, whose height, weight, and pubertal stages were measured and assessed by pediatricians. BMI standard deviation scores (BMISDS) were used to categorize children as underweight (BMISDS < −2); normal-weight (−2 ≤ BMISDS ≤ 1); overweight (1 < BMISDS ≤ 2); and obese (BMISDS > 2). Children were divided into low-level (<−0.67 SD) and nonlow-level (≥−0.67 SD) groups based on IGF-1 standard deviation scores (IGF-1SDS). The association between IGF-1 and BMI as categorical and continuous variables was explored by Binary logistic regression, the restrictive cubic spline model, and the generalized additive model. Models were adjusted by height and pubertal development. Recursive algorithm and multivariate piecewise linear regression were further utilized to assess the threshold of the smooth curve. Results: IGF-1 levels varied by BMI categories, with the highest levels observed in the overweight group. The proportion of low IGF-1 levels in underweight, normal-weight, overweight, and obese groups was 32.1%, 14.2%, 8.4%, and 6.5%, respectively. The risk odds of low IGF-1 levels in underweight children were 2.86-, 2.20-, and 2.25-fold higher than in children with normal weight before adjustment, after adjustment for height, and after adjustment for height and puberty, respectively. When analyzing the association between BMI and low IGF-1 levels, dose-response analysis demonstrated an inverted J-shaped relationship between BMISDS and low IGF-1 levels. Lower or higher BMISDS increased the odds of low IGF-1 levels, and significance was retained in underweight children but not in obese children. When BMI and IGF-1 levels were used as continuous variables, the relationship between the BMISDS and IGF-1SDS followed a nonlinear inverted U shape. IGF-1SDS increased with the increase of BMISDS (*β* = 0.174, 95% CI: 0.141 to 0.208, *p* < 0.01) when BMISDS was less than 1.71 standard deviation (SD) and decreased with the increase of BMISDS (*β* = −0.358, 95% CI: −0.474 to −0.241, *p* < 0.01) when BMISDS was greater than 1.71 SD. Conclusions: The relationship between BMI and IGF-1 levels was found to depend on the type of variable, and extremely low or high BMI values could result in a tendency toward low IGF-1 levels, emphasizing the importance of maintaining a normal BMI range for normal IGF-1 levels.

## 1. Introduction

Insulin-like growth factor-1 (IGF-1) is an essential mediator of growth hormone (GH), which is involved in normal metabolic homeostasis and physical growth [1,2,3,4]. In children, IGF-1 levels are positively associated with physical growth, bone mass, puberty initiation, and pubescent growth spurt [5,6,7,8,9]. IGF-1 levels are crucial in the clinical diagnosis and monitoring of GH-related diseases in children with growth retardation or acromegalic [1,2,3,4], and can be affected by various factors, such as gender, age, disease, pubertal development, and nutritional status [2,10].

Nutrition is a key factor affecting IGF-1 synthesis and secretion, and IGF-1 levels are dysregulated in the condition of undernutrition or overnutrition [4,11], but the relationship between body mass index (BMI) and IGF-1 levels remains unclear. While it is well established that IGF-1 levels are reduced in underweight children, evidence of the association between obesity and IGF-1 levels has been inconclusive. Previous relevant investigations have suggested that there was no significant difference in IGF-1 levels between obese and normal-weight children [12,13]. In contrast, other research suggested that obese children have higher [6,10,14] or lower [15,16,17] IGF-1 levels than nonobese children. The likely reason may be that previous studies have mostly focused on comparing IGF-1 levels between different nutritional groups based on BMI categories, disregarding the continuous changes in BMI and IGF-1 levels, as well as confounding factors such as gender, age, height, and pubertal development.

Obesity is a significant risk factor for endocrine and cardiovascular chronic diseases, and recent studies have demonstrated that low IGF-1 levels in obese children are closely related to nonalcoholic fatty liver disease, low high-density lipoprotein cholesterol, and insulin resistance [15,18,19]. Therefore, examining the relationship between nutritional status and IGF-1 levels has significant implications for individualized assessment and monitoring of overweight and obese children and may also provide new evidence-based clues and implications for alleviating obesity-associated complications and preventing chronic diseases.

To fill this knowledge gap, this study aimed to examine the continuous changes in BMI and IGF-1 levels and further examine the impact of being underweight, being overweight, and obesity on IGF-1 levels in Chinese children aged 2–18 years.

## 2. Methods

### 2.1. Setting

Capital Institute of Pediatrics, Beijing, China.

### 2.2. Study Design

This cross-sectional survey was approved by the Ethics Committee of the Capital Institute of Pediatrics (ethics approval number: SHERLL 2016070). The research assistant explained the applications of clinical data to the guardians and/or children and obtained their consent before collecting blood samples.

### 2.3. Participants

Participants were recruited from the Department of Growth and Development Outpatient Clinic of Children’s Hospital, Affiliated to the Capital Institute of Pediatrics (Beijing, China) between January 2017 and March 2022. Participants were confirmed to be in relatively good health without specific diseases by physical examination and laboratory testing.

The exclusion criteria for this study were: (1) incomplete clinical medical records; (2) children with acute or chronic diseases, especially those with liver and kidney disease, severe heart disease, and endocrine metabolic disease, or underweight and obese children with determined etiology; (3) children with congenital abnormalities, such as skeletal dysplasia or disorders of sex development; and (4) children with a history of receiving medications (exogenous hormones or drugs) that may affect IGF-1 levels and body weight.

Participant data were collected, including gender, age, height, weight, BMI, pubertal development, and serum-free IGF-1 levels.

### 2.4. Physical Examination

Physical examinations were conducted following standardized procedures.

Height and weight were measured by pediatricians; before the measurement, children were instructed to wear light clothing without shoes or hats. Standing height was measured with a human altimeter (Seca213, Seca GmbH & Co. KG, Hamburg, Germany) and weight with an electronic scale (JT-918, J-Sky Juding Tianheng (Suzhou) Weighing Equipment Co., Ltd., Suzhou, China), with a precision of 0.1 cm and 0.1 kg, respectively. BMI = weight (kg)/height (m)^2^. Height, weight, and BMI were converted into height standard deviation scores (HtSDS), weight standard deviation scores (WtSDS), and BMI standard deviation scores (BMISDS) based on Chinese children and adolescent growth charts. The Lambda Mu and Sigma method (LMS) was used to calculate SDS; SDS = [(X/M)^L^ − 1]/LS (X: Measurement, M: Median, L: Box-C0c transformation, S: the generalized coefficient of variation) [20]. Nutritional status was assessed based on BMISDS, and the children were classified into underweight (BMISDS < −2), normal (−2 ≤ BMISDS ≤ 1), overweight (1 < BMISDS ≤ 2), and obese (BMISDS > 2) groups, according to the cut-off values recommended by the World Health Organization.

Pubertal status was determined by pediatricians using breast and pubic hair in girls [21] or testicular and pubic hair in boys [22], based on the Tanner stages. Children in Tanner stage I were classified as prepubescent, and those who had already reached stages II–V were classified as pubescent.

### 2.5. Laboratory Measurement

Fasting blood was taken in the morning, and serum IGF-1 concentration was tested using the chemiluminescence method (IMMULITE/IMMULITE1000 IGF-1; Siemens Healthcare Diagnostics, Glyn Rhonwy, Llanberis, UK). The analytical sensitivity for IGF-1 was 20 ng/mL [23]. Since IGF-1 levels are affected by age and gender, we standardized the two indicators by age and gender to eliminate the differences when analyzing the relationship between BMI and IGF-1. IGF-1 standard deviation scores (IGF-1SDS) were calculated based on the reference standard in the kit. As IGF-1 followed an abnormal distribution, the 25th percentile (P25) was used as the cut-off point to reclassify children into low levels (IGF-1SDS < −0.67) and nonlow levels (IGF-1SDS ≥ −0.67) groups.

### 2.6. Statistical Analysis

The EpiData version 3.1 (The EpiData Association, Odense, Denmark) was used to establish the database, and statistical analysis and graph drawing were performed using R version 4.2.0 (https://www.R-project.org, accessed on 5 April 2022) and RStudio (https://www.rstudio.com, accessed on 5 April 2022). Continuous data were expressed as means (SD), and categorical data as numbers (proportion). Analysis of variance (ANOVA) with least significant difference (LSD) post hoc was used for normally distributed data to compare differences between different BMI categories, while Kruskal–Wallis, followed by Dunnett’s T3 post hoc test was used for non-normally distributed data. The Chi-square test was used for categorical data. The relationship between IGF-1 levels and BMI categories was explored using a binary logistic regression model, with results presented as odds ratios (OR) and 95% confidence intervals (CI). To control for potential confounders, three models were conducted in the analyses: Model 1 was without adjustment, Model 2 was adjusted for height, and Model 3 was adjusted for height and pubertal development. The dose-response relationship between BMISDS (as a continuous variable) and low IGF-1 levels was assessed by restricted cubic spline (RCS) logistic regression models with adjustment for height and pubertal development. The generalized additive model (GAM) was performed to fit the smooth curve to identify the continuous variation between BMISDS and IGF-1SDS, adjusted for height and pubertal development. Recursive algorithm and multivariate piecewise linear regression were further utilized to assess the threshold of the smooth curve. Statistical significance was considered at *p* < 0.05.

## 3. Results

A total of 3227 children participated in this study, comprising 1447 boys (44.8%) and 1780 girls (55.2%). The mean age of boys was 9.0 (standard deviation or SD: 3.3) years, and that of girls was 8.5 (SD: 2.3) years. Among them, 84 (2.6%), 524 (16.2%), and 278 (8.6%) were categorized into underweight, overweight, and obese groups, respectively.

### 3.1. Demographic and Anthropometric Characteristics

Table 1 summarizes the demographic and physical growth characteristics of the participants in the different nutritional groups. The analysis revealed that IGF-1 levels initially increased and then decreased with the BMI categories, with the highest levels observed in the overweight group. The underweight group showed significantly lower IGF-1SDS compared to other groups (*p* < 0.01), and the average IGF-1SDS in underweight, normal-weight, overweight and obese groups were −0.2 (SD: 0.8), 0.3 (SD: 1.0), 1.1 (SD: 1.4), and 1.0 (SD: 1.5), respectively. The proportion of low IGF-1 levels was found to be highest in the underweight group at 32.1%, while it was 14.2%, 8.4%, and 6.5% for the normal-weight, overweight, and obese groups, respectively.

### 3.2. Associations of BMI Categories with IGF-1

Table 2 displays the results of the logistic regression analysis on the association between BMI categories and the risk of low IGF-1 levels. The analysis revealed that underweight children had a significantly higher risk of low IGF-1 levels compared to those with normal weight, with ORs of 2.86 (95% CI: 1.79, 4.59), 2.20 (95% CI: 1.33, 3.65), and 2.25 (95% CI: 1.36, 3.71) after adjusting for no confounder, height, and height and pubertal development, respectively. On the other hand, being overweight and obese were initially found to be protective factors against low IGF-1 levels, but this effect disappeared after controlling for height and pubertal development. After adjusting for no confounder, height, and height and pubertal development, the ORs for low IGF-1 levels were 0.56 (95% CI: 0.40, 0.77), 1.12 (95% CI: 0.78, 1.59), and 1.15 (95% CI: 0.81, 1.65) in the overweight group, and 0.42 (95% CI: 0.26, 0.69), 1.43 (95% CI: 0.82, 2.47), and 1.15 (95% CI: 0.88, 2.65) in the obese group, respectively.

### 3.3. Nonlinear Relationship of BMISDS with IGF-1SDS

**Figure 1:**
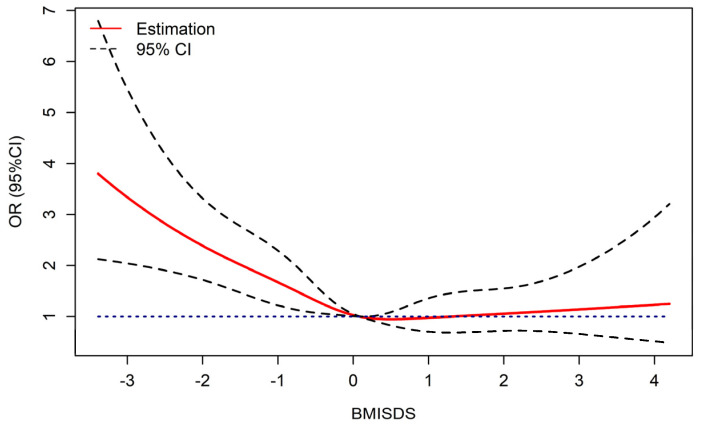
Dose-response relationship of BMISDS with low IGF-1 levels (adjusted for pubertal development and height standard deviation scores). OR: odds ratios, CI: confidence intervals, BMISDS: body mass index standard deviation scores, IGF-1: insulin-like growth factor-1. Blue dashed line represents OR = 1.

Figure 1 displays the dose-response relationships between BMISDS and low IGF-1 levels, with an inverse J-shaped curve after adjustment for height and pubertal development. The RCS logistic regression models demonstrated a nonlinear association between BMISDS and low IGF-1 levels (*P*_nonlinear_ = 0.0114). Lower or higher BMISDS increased the odds of low IGF-1 levels, significance was retained in underweight children, but no significance was noted in obese children.

**Figure 2:**
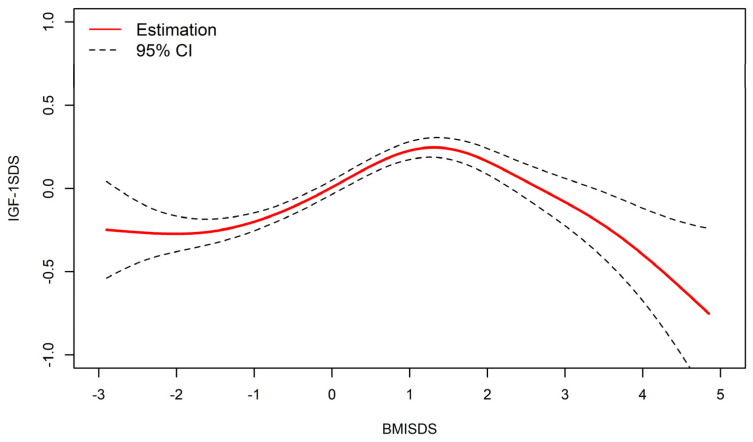
Nonlinear relationship between BMISDS and IGF-1SDS (adjusted for pubertal development and height standard deviation scores). BMISDS: body mass index standard deviation scores, IGF-1SDS: insulin-like growth factor-1standard deviation scores; CI: confidence intervals. R^2^ = 0.416.

As shown in Figure 2, after adjusting for height and pubertal development, an inverted U-shaped relationship between BMISDS and IGF-1SDS existed, with a threshold point observed in the resultant smooth curve.

The threshold point of the curve was determined to be 1.71 SD (P95.8), IGF-1 levels increased in BMISDS lower than 1.71 SD (*β* = 0.174, 95% CI: 0.141, 0.208, *p* < 0.01), with progressively lower values thereafter (*β* = −0.358, 95%CI: −0.474, −0.241, *p* < 0.01) (Table 3).

## 4. Discussion

The key finding of this study is the existence of a nonlinear relationship between BMI and IGF-1 levels. Specifically, an inverted J-shaped association between BMISDS and low IGF-1 levels was observed. When BMISDS and IGF-1SDS were used as continuous variables, they showed an inverted U-shaped relationship, with the highest levels at 1.71 SD, and the IGF-1 levels showed a tendency to decrease in underweight children or children with extremely high BMI values. These findings highlight the importance of BMI for normal IGF-1 levels and provide clarification on previously controversial results.

Nutritional status has a significant impact on IGF-1 levels [4]. The present study found that underweight children were prone to low IGF-1 levels (32.1%), which may be due to decreased protein and energy intake. Maintenance of IGF-1 levels requires an adequate energy supply. A study published by Snyder et al. [24] showed that adults need to consume at least 700 kcal of carbohydrates per day to maintain optimal levels of IGF-1. In the case of starvation, semistarvation, fasting, and protein or calorie restriction, metabolic resources used for growth are diverted to meet the immediate energy needs of individuals, resulting in decreasedIGF-1 levels [25,26]. This can further promote protein catabolism in skeletal muscle [25] and result in impaired height growth. The study also found that the height of underweight children was the lowest. In addition, the relative resistance to GH and the decreased expression of GH receptors during malnutrition are also the reasons for the decreased IGF-1 level [25]. Thus, early nutritional intervention, improvement of feeding and eating habits, returning to normal weight, and maintenance of IGF-1 levels at a normal range may be effective at improving malnourished growth retardation or growth failure in underweight children.

The relationship between obesity and IGF-1 levels remains a topic of controversy, as different studies have reported higher [6,10,14], lower [15,16,17], or comparable [12,13] IGF-1 levels in obese children compared to nonobese children. The inconsistency in previous findings may be attributed to differences in sample size, age, race, pubertal stages, and the degree of obesity. Relatively few studies have investigated the dose-response relationships and continuity changes between BMI and IGF-1 levels. Consistent results can be obtained through the present study, that is, there is a nonlinear relationship, but the shapes of the curves are different. There was an inverted J-shaped dose-response relationship between BMISDS and low-level IGF-1 after adjustment for potential confounding factors. However, when BMISDS and IGF-1 were used as continuous variables, the relationships were inverted U-shaped after adjustment for potential confounding factors, with an optimal threshold point of 1.71 SD (P95.8), approximately equivalent to the critical point of obesity. This is consistent with a previous German study, which reported that compared to adults with BMIs between 20 and 35 kg/m^2^, the IGF-1 levels decreased when BMI < 20 or >35 kg/m^2^ [27]. These findings partially support the previous findings of some scholars and highlight the importance of BMI for normal IGF-1 levels. The mechanism underlying decreased IGF-1 levels in children with extremely high BMI values remains unclear, but possible reasons include: (1) A greater mass of adipose tissue could affect IGF-1 production in the liver [28]. (2) Elevated estrogen levels in obese children inhibit the action of GH on the liver, thereby decreasing the production of IGF-1 [29]. (3) The IGF-1 levels negatively correlated with the levels of inflammatory cytokines. The secretion of large quantities of inflammatory cytokines (such as TNF-α, IL-6) in adipose tissues could affect the GH release and actions and inhibit the secretion of IGF-1 [28].

Our study results indicate limitations in the role of IGF-1 levels in identifying GH/IGF-1-related disease in underweight and obese children. While IGF-1 is commonly used as an indicator to screen for GH deficiency in children with growth failure or short stature, our findings suggest that IGF-1 levels may be less accurate for detecting GH deficiency in underweight and obese children, which may result in misdiagnosis. Moreover, decreased IGF-1 levels in children with extremely high BMI values may negatively affect pubertal growth. A previous cohort study conducted in Sweden revealed that for each 1-unit increase in BMI between the ages of 2–8 years, pubertal height growth could decrease by 0.88 cm in boys and 0.51 cm in girls [30]. Although adult height may not be affected in obese children due to prepubertal growth advantage, impaired adult height cannot be ruled out in obese children who are short or relatively short before puberty.

The study provides new insights into the relationship between obesity and related chronic diseases, particularly the potential role of IGF-1 levels as an early warning indicator. Emerging evidence suggests that low IGF-1 levels in obese individuals may increase the risk of nonalcoholic fatty liver disease, metabolic syndrome, and insulin resistance [15,18,19]. In a GH-deficient rat model, GH-independent IGF-I action was found to play an important role in the liver and prevent the development of nonalcoholic steatohepatitis [31]. It is not known whether low IGF-1 levels are also associated with an increased incidence of chronic diseases in adulthood, such as diabetes, hypertension, and coronary heart disease, in chronically malnourished children [32]. Thus, maintaining IGF-1 levels in the normal range contributes to healthy adulthood and may reduce the incidence of chronic diseases. It is worth noting that the decrease in IGF-1 levels caused by being underweight and obesity can be reversed by improving nutrition. A previous study reported that IGF-1 levels increased after weight gain in underweight children, while the IGF-1 levels significantly increased after weight loss of obese children over 25.0%, approaching levels seen in normal-weight children [33]. Therefore, it is essential to pay more attention to the negative effects of low IGF-1 levels and carry out comprehensive weight management and intervention in underweight and obese children as early as possible. Our exploratory findings provide a potential avenue for further research into obesity-related chronic diseases and may provide new approaches for the treatment of obesity-related pathological conditions.

The present study has several strengths, including a relatively large sample size and the use of both categorical and continuous BMI data to explore the relationship between nutritional status and IGF-1 levels. Our results showed that when BMI was used as a categorical variable, IGF-1 levels decreased in underweight children and increased in overweight and obese children. Furthermore, when BMI was utilized as a continuous variable, BMISDS and IGF-1SDS showed an inverted U-shaped nonlinear relationship after adjusting for confounding factors. To our knowledge, this is the first study to investigate continuous changes between BMI and IGF-1 levels in children, providing new insights into the relationship between nutritional status and IGF-1 levels. However, some limitations of the study should be acknowledged. First, the study did not include data on insulin-like growth factor binding proteins (IGFBPs), which prevented the analysis of the changes in the ratio of IGF-1 to IGFBP3. Future research should address this issue. Second, the study did not collect data on different types of obesity, making it difficult to determine the relationship between different types of obesity and IGF-1 levels. Thus, further investigation is needed to explore the relationship among BMI, body composition, IGF-1 levels, and IGFBPs levels. Finally, as a cross-sectional study, the findings of the present study cannot establish causality between BMI and IGF-1 levels. Future research should use longitudinal data and representative cohorts to investigate this relationship in more depth.

## 5. Conclusions

Our study identified a nonlinear relationship between BMI and IGF-1 levels, with an inverted J-shaped association between BMISDS and low IGF-1 levels, and an inverted U-shaped relationship between BMISDS and IGF-1SDS. Our results suggest that maintaining a normal BMI range is crucial for normal IGF-1 levels, as underweight children and those with extremely high BMIs tended to have decreased IGF-1 levels. However, further research investigating dose-response associations and continuous variations in this area is needed to fully understand the relationship between BMI and IGF-1 levels.

## Figures and Tables

**Table 1:** Characteristics of participants by nutritional groups.

Variables		Total	Underweight	Normal	Overweight	Obese	*p*
N (%)		3227	84 (2.6)	2341 (72.5)	524 (16.2)	278 (8.6)	
Gender, *n* (%)							
Boys		1447 (44.8)	50 (3.5)	1091 (75.4)	200 (13.8)	106 (7.3)	<0.01
Girls		1780 (55.2)	34 (1.9)	1250 (70.2)	324 (18.2)	172 (9.7)	
Age, years, mean (SD)		8.8 ± 2.8	8.9 ± 3.2	8.6 ± 2.8	9.3 ± 2.5	9.4 ± 2.4	<0.01
(min, max)		(2.0, 17.6)	(2.8, 14.7)	(2.0, 17.6)	(2.4, 16.2)	(2.4, 15.6)	
Height, cm, mean (SD)		129.8 ± 18.3	126.0 ± 18.4	127.2 ± 18.0	136.5 ± 17.2	140.4 ± 14.6	<0.01 ^a^
(min, max)		(81.6, 186.0)	(87.2, 164.3)	(80.5, 184.0)	(82.5, 181.0)	(83.5, 186.0)	
HtSDS, mean (SD)		−0.6 ± 1.4	−1.4 ± 0.9	−0.9 ± 1.2	0.1 ± 1.4	0.7 ± 1.5	<0.01
(min, max)		(−2.8, 5.1)	(−2.8, 1.9)	(−2.4, 3.8)	(−2.1, 4.9)	(−2.3, 5.1)	
Weight, mean (SD)		30.1 ± 12.5	21.3 ± 6.8	26.4 ± 9.3	38.3 ± 12.6	47.9 ± 14.2	<0.01
(min, max)		(10.0, 90.0)	(10.0, 37.4)	(10.2, 70.0)	(12.5, 85.0)	(13.0, 90.0)	
WtSDS, mean (SD)		−0.2 ± 1.4	−2.3 ± 0.6	−0.7 ± 0.9	1.0 ± 0.8	2.3 ± 1.0	<0.01
(min, max)		(−3.4, 6.1)	(−3.4, −0.5)	(−2.4, 2.1)	(−1.3, 3.0)	(−1.4, 6.1)	
BMI, mean (SD)		17.0 ± 3.2	13.0 ± 0.7	15.8 ± 1.7	19.8 ± 2.3	23.7 ± 3.2	<0.01
(min, max)		(11.8, 38.9)	(11.8, 15.1)	(12.6, 23.6)	(16.5, 26.9)	(18.3, 38.9)	
BMISDS, mean (SD)		0.2 ± 1.2	−2.3 ± 0.2	−0.3 ± 0.7	1.5 ± 0.3	2.6 ± 0.6	<0.01
(min, max)		(−2.9, 4.9)	(−2.9, −2.1)	(−2.0, 1.0)	(1.1, 2.0)	(2.0, 4.9)	
Pubertal stages, *n* (%)							
Prepubescent	Stage I	1444 (44.7)	51 (3.5)	1205 (83.4)	135 (9.3)	53 (3.7)	<0.01
Pubescent	Stage II	1027 (31.8)	30 (2.9)	739 (72.0)	171 (16.7)	87 (8.5)	
	Stage III	507 (15.7)	3 (0.6)	279 (55.0)	145 (28.6)	80 (15.8)	
	Stage IV	213 (6.6)	0	99 (46.5)	64 (30.0)	50 (23.5)	
	Stage V	36 (1.1)	0	19 (52.8)	9 (25.0)	8 (22.2)	
IGF-1, mean (SD)		226.5 ± 133.8	169.0 ± 89.4	207.2 ± 122.7	293.3 ± 150.8	281.1 ± 143.8	<0.01 ^b^
(min, max)		(25.0, 940.0)	(25.0, 405.0)	(25.0, 795.0)	(27.1, 940.0)	(31.9, 796.0)	
IGF-1SDS, mean (SD)		0.5 ± 1.2	−0.2 ± 0.8	0.3 ± 1.0	1.1 ± 1.4	1.0 ± 1.5	<0.01 ^c^
(min, max)		(−1.6, 5.7)	(−1.4, 3.2)	(−1.5, 5.5)	(−1.6, 5.7)	(−1.5, 5.2)	
IGF-1 levels group, *n* (%)							
Low levels		422 (13.1)	27 (32.1)	333 (14.2)	44 (8.4)	18 (6.5)	<0.01
Nonlow levels		2805 (86.9)	57 (67.9)	2013 (85.8)	478 (91.6)	257 (93.5)	

HtSDS: height standard deviation scores; SD: standard deviation; WtSDS: weight standard deviation scores; BMISDS: BMI standard deviation scores; IGF-1: insulin-like growth factor-1; IGF-1SDS: insulin-like growth factor-1standard deviation scores. ^a^: no significance was noted in the post hoc test between the underweight group and the normal group (*p* = 0.986); ^b^: no significance was noted in the post hoc test between the underweight group and the normal group (*p* = 0.06), or between the overweight group and the obesity group (*p* = 0.844); ^c^: no significance was noted in the post hoc test between the overweight group and the obesity group (*p* = 0.9).

**Table 2:** Individual prediction of BMI categories for low IGF-1 levels.

BMI Categories	OR (95% CI)		
	Model 1	Model 2	Model 3
Underweight (BMISDS < −2)	2.86 (1.79, 4.59)	2.20 (1.33, 3.65)	2.25 (1.36, 3.71)
Normal-weight (−2 ≤ BMISDS ≤ 1)	Reference	Reference	Reference
Overweight (1< BMISDS ≤ 2)	0.56 (0.40, 0.77)	1.12 (0.78, 1.59)	1.15 (0.81, 1.65)
Obese (BMISDS > 2)	0.42 (0.26, 0.69)	1.43 (0.82, 2.47)	1.52 (0.88, 2.65)

BMI: body mass index; BMISDS: IGF-1: insulin-like growth factor-1; BMI standard deviation scores; OR: odds ratio; CI: confidence interval. Model 1: without adjustment; Model 2: adjustment for height; Model 3: adjustment for height and pubertal development.

**Table 3:** Threshold effects of IGF-1SDS with BMISDS were analyzed using linear regression.

Threshold Point of BMISDS	*β* (95% CI)	*p*
<1.71 (P95.8)	0.174 (0.141, 0.208)	<0.01
≥1.71 (P95.8)	−0.358 (−0.474, −0.241)	<0.01

BMISDS: BMI standard deviation scores; IGF-1SDS: insulin growth factor-1 standard deviation scores; CI: confidence intervals.

## Data Availability

The data used in the present study are available on request from the corresponding author. The data are not publicly available for privacy considerations.
